# Targeting carbonic anhydrase IX improves the anti-cancer efficacy of mTOR inhibitors

**DOI:** 10.18632/oncotarget.9134

**Published:** 2016-05-02

**Authors:** Seraina Faes, Anne Planche, Emilie Uldry, Tania Santoro, Catherine Pythoud, Jean-Christophe Stehle, Janine Horlbeck, Igor Letovanec, Nicolo Riggi, Dipak Datta, Nicolas Demartines, Olivier Dormond

**Affiliations:** ^1^ Department of Visceral Surgery, University Hospital and University of Lausanne, Lausanne, Switzerland; ^2^ Mouse Pathology Facility, University Hospital and University of Lausanne, Lausanne, Switzerland; ^3^ Institute of Pathology, University Hospital and University of Lausanne, Lausanne, Switzerland; ^4^ Biochemistry Division, CSIR-Central Drug Research Institute, Lucknow, India; ^5^ Academy of Scientific and Innovative Research, New Delhi, India

**Keywords:** mTOR, CAIX, hypoxia, rapamycin, acetazolamide

## Abstract

The inhibition of the mechanistic target of rapamycin complex 1 (mTORC1) by chemical inhibitors, such as rapamycin, has demonstrated anti-cancer activity in preclinical and clinical trials. Their efficacy is, however, limited and tumors eventually relapse through resistance formation. In this study, using two different cancer mouse models, we identify tumor hypoxia as a novel mechanism of resistance of cancer cells against mTORC1 inhibitors. Indeed, we show that the activity of mTORC1 is mainly restricted to the non-hypoxic tumor compartment, as evidenced by a mutually exclusive staining pattern of the mTORC1 activity marker pS6 and the hypoxia marker pimonidazole. Consequently, whereas rapamycin reduces cancer cell proliferation in non-hypoxic regions, it has no effect in hypoxic areas, suggesting that cancer cells proliferate independently of mTORC1 under hypoxia. Targeting the hypoxic tumor compartment by knockdown of carbonic anhydrase IX (CAIX) using short hairpin RNA or by chemical inhibition of CAIX with acetazolamide potentiates the anti-cancer activity of rapamycin. Taken together, these data emphasize that hypoxia impairs the anti-cancer efficacy of rapalogs. Therapeutic strategies targeting the hypoxic tumor compartment, such as the inhibition of CAIX, potentiate the efficacy of rapamycin and warrant further clinical evaluation.

## INTRODUCTION

Over the last decade, extensive research focused on targeting signaling pathways that are deregulated in cancer and promote tumor growth. In this context, blocking the mechanistic target of rapamycin (mTOR) has demonstrated clinical benefits in cancer patients that are however limited due to the development of resistance mechanisms [1–4]. Hence, it is important to identify these mechanisms in order to improve the efficacy of mTOR inhibitors. mTOR exerts its biological functions as a subunit of two different protein complexes; the mTOR complex 1 (mTORC1) and the mTOR complex 2 (mTORC2) [5]. mTORC1 is activated by growth-promoting stimuli such as growth factors or amino acids. In contrast, unfavorable growth conditions such as low energy levels or hypoxia lead to mTORC1 inactivation. Once activated, mTORC1 controls cell growth through various mechanisms including protein, lipid and nucleotide synthesis as well as inhibition of autophagy [5, 6]. In the context of cancer, overactivation of mTORC1 is frequently observed in human tumors caused either by activating mutations of upstream components of the mTOR signaling pathway or by mutations of mTOR itself [7, 8]. Accordingly, several preclinical and clinical studies have evaluated the anti-cancer efficacy of mTORC1 inhibition by the chemical inhibitor rapamycin and its analogs termed rapalogs. Following encouraging results in mouse models, the efficacy of rapalogs was however lower than expected in patients [3].

Regions of hypoxia are frequently present in tumors and profoundly influence the biology of cancer and its response to therapies [9, 10]. Classically, a high rate of cancer cell proliferation combined with structural abnormalities of tumor endothelial cells induces regions in tumors featuring low oxygen levels. Tumor cells are able to quickly adapt to this hypoxic microenvironment by inducing the transcriptional activity of hypoxia inducible factors [11]. Among the different proteins whose expressions are stimulated by hypoxia, emerging evidence point out the importance of the carbonic anhydrase IX (CAIX) enzyme in promoting cancer cell growth [12]. Indeed, blocking CAIX either by genetic manipulations or by using chemical inhibitors significantly reduces the growth of tumor xenografts in mice [13–15]. Moreover, targeting CAIX enhances the anti-cancer efficacy of anti-angiogenic therapies or radiotherapy [14, 16]. Furthermore, expression of CAIX in human tumor samples is associated with tumor progression and poor prognosis [17, 18].

In this study, we found that mTORC1 activity was diminished in hypoxic tumor regions. Consequently, rapamycin failed to reduce cancer cell proliferation in these regions. We further observed that combining treatments that target components of the hypoxic tumor response, such as CAIX, potentiated the anti-cancer efficacy of rapamycin. Taken together, these results show that hypoxic cancer cells proliferate independently of mTORC1 and are hence intrinsically resistent to mTOR inhibitors. They further provide a rationale to combine CAIX and mTOR inhibitors in cancer therapy.

## RESULTS

### mTORC1 activity in cancer cells is reduced in hypoxic regions of the tumor

**Figure 1:**
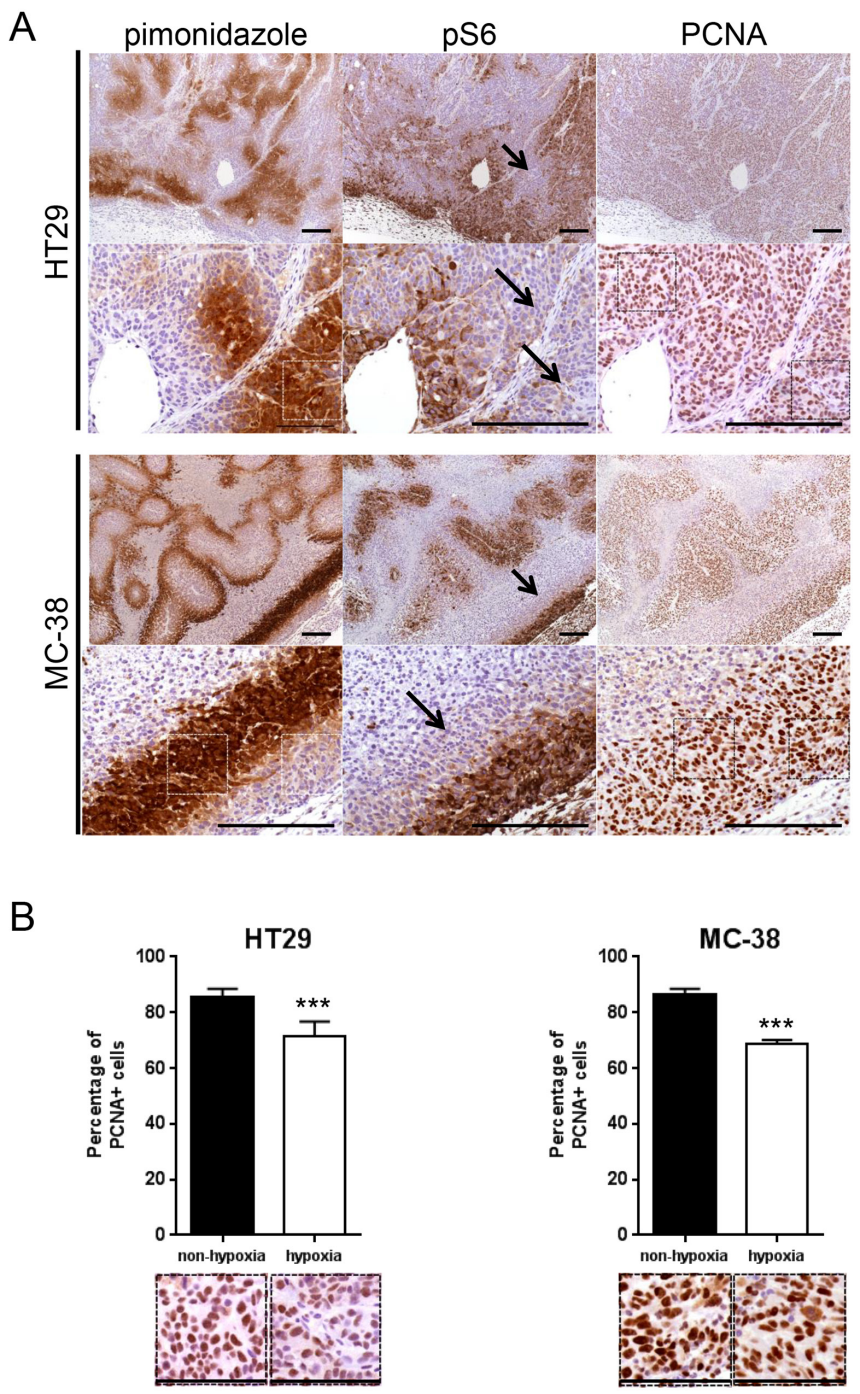
mTORC1 activity is reduced in hypoxic regions of a tumor **A.** Serial sections of HT29 tumor xenografts and MC-38 tumor allografts were stained for pimonidazole, pS6 or PCNA. Arrows point to pimonidazole positive, pS6 negative regions. Scale bar, 200 μm. **B.** Percentage of PCNA positive cancer cells was counted in 10 representative pimonidazole positive and 10 representative pimonidazole negative zones of a 100 × 100 μm surface for three different HT29 and MC-38 tumors for a total of 30 pimonidazole positive and 30 pimonidazole negative zones for each cell line (1 pimonidazole positive and 1 pimonidazole negative area used for counting is highlighted by squares (white in pimonidazole staining and black in PCNA staining) under A and displayed under B). Bar charts represent mean, error bars represent SD. *** p<0.001, Student's t-test. Representative image section below corresponding bar chart, scale bar, 100 μm.

It was previously reported that hypoxia inhibits mTORC1 activity *in vitro* [19]. We therefore first hypothesized that mTORC1 function is mainly present in non-hypoxic areas of a tumor. To test this, human colorectal adenocarcinoma cell line HT29 xenografts were generated in nude and murine colon adenocarcinoma cell line MC-38 allografts in C57BL/6 mice. After tumor harvest, mTORC1 activity and hypoxia were detected using immunohistochemical staining for phospho-S6 ribosomal protein (pS6) and pimonidazole respectively. In addition, proliferating cell nuclear antigen (PCNA) staining was applied to assess cancer cell proliferation. We found that pS6 and pimonidazole stainings negatively correlated, whereas PCNA staining revealed proliferation in both compartments (Figure 1A). Similarly, the staining of phospho-4E-BP1, another downstream target of mTORC1, was predominantly found in pimonidazole negative tumor areas (Supplementary Figure S1). This suggests that, in hypoxic zones, cancer cells proliferate despite the reduction of mTORC1 activity (Figure 1A and 1B). Proliferation rate was however significantly decreased in hypoxic compared to non-hypoxic regions (proliferation rate in hypoxic region: HT29 71.4 %, MC-38 68.9 %; proliferation rate in non-hypoxic region: HT29 85.7 %, MC-38 86.4 %) (Figure 1B).

### Rapamycin selectively reduces proliferation of cancer cells in non-hypoxic zones of tumors

**Figure 2:**
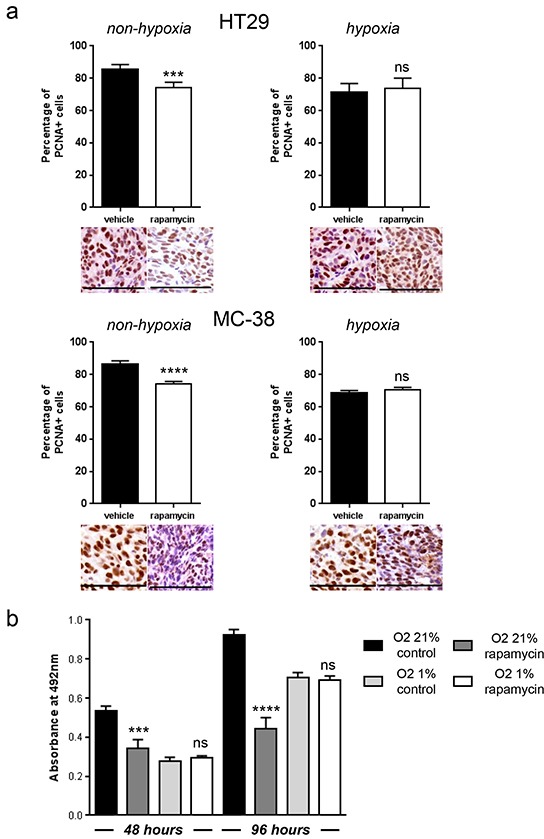
Hypoxic tumor regions proliferate independently of mTORC1 and are resistant to rapamycin **A.** Percentage of PCNA positive cancer cells was counted in 10 representative pimonidazole positive and pimonidazole negative zones of a 100 × 100 μm surface for three different HT29 and MC-38 tumors. Representative image section below corresponding bar chart, scale bar, 100 μm. **B.** MTS cell proliferation assay of HT29 cells cultured in hypoxia (O_2_ 1 %) or non-hypoxia (O_2_ 21 %) and treated with DMSO or rapamycin 100 nM was performed after 48 and 96 hours. Bar charts represent mean, error bars represent SD. **** p<0.0001, *** p<0.001, ns=not significant, Student's t-test.

**Figure 3:**
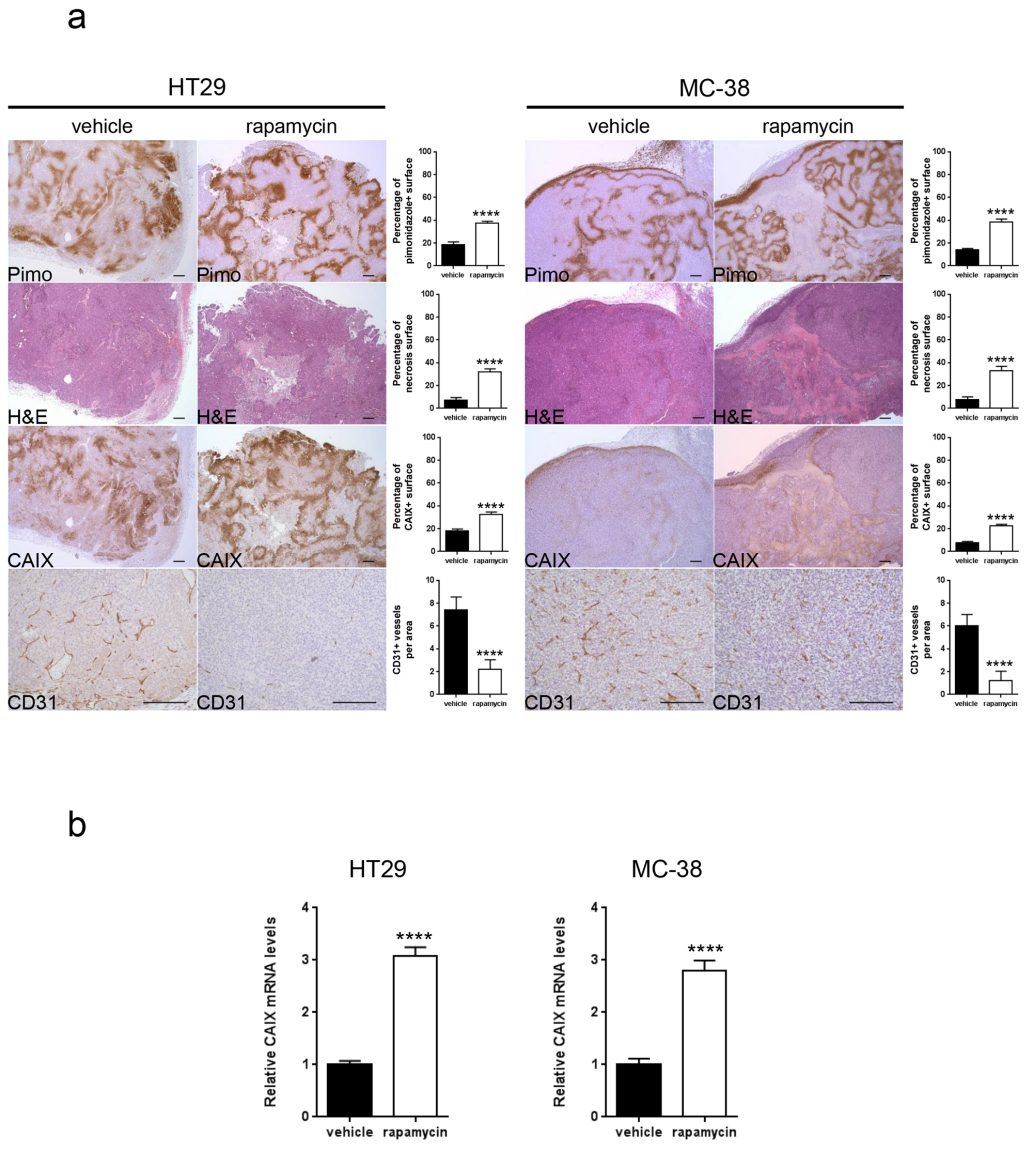
Rapamycin increases carbonic anhydrase IX expression, tumor hypoxia and tumor necrosis and decreases tumor vasculature **A.** Percentage of tumor hypoxia (pimonidazole positive surface), tumor necrosis (light pink stained surface in H&E) and CAIX expression (CAIX positive surface) were compared for vehicle and rapamycin treated tumors of HT29 xenograft and MC-38 allograft in 10 representative sections of 3368 × 2668 μm for three different tumors. Tumor vasculature was analyzed by counting CD31 positive vessels in 10 representative sections of 200 × 200 μm for three different tumors. Scale bars, 200 μm. **B.** mRNA was extracted from 5 tumors and tested for CAIX levels and GAPDH as a control by qRT- PCR. Bar charts represent mean, error bars represent SD. **** p<0.0001, Student's t-test.

We next hypothesized that, since mTORC1 activity is reduced in hypoxic regions, blocking mTOR with rapamycin would not influence cancer cell proliferation in these regions. To test this, nude mice bearing HT29 tumor xenografts or C57BL/6 mice bearing MC-38 allografts were treated with rapamycin or vehicle as a control. We found that, whereas rapamycin significantly reduced tumor cell proliferation in non-hypoxic zones (HT29: vehicle 85.7 %, rapamycin 74.3 %; MC-38: vehicle 86.4 %, rapamycin 74.2 %), it had no effect on hypoxic areas (HT29: vehicle 71.4 %, rapamycin 73.6 %; MC-38: vehicle 68.9 %, rapamycin 70.5 %) (Figure 2A). In accordance with these *in vivo* results, we found no anti-proliferative effect of rapamycin on cancer cells cultured in hypoxic conditions (1 % oxygen) (Figure 2B). Interestingly, *in vivo*, rapamycin increased the hypoxic tumor compartment compared to controls in both HT29 tumor xenografts (from 18.5 % to 37.6 %) and MC-38 tumor allografts (from 14.0 % to 38.2 %) (Figure 3A). Consistent with this, we observed that rapamycin increased the level of CAIX protein expression from 18.2 % to 32.4 % in HT29 tumors and from 7.8 % to 22.8 % in MC-38 tumors. This increased expression of CAIX was associated with a reduction of CD31 positive tumor blood vessels (Figure 3A). Upregulation of CAIX levels in the treated xeno- and allografts was confirmed by qRT-PCR (Figure 3B).

### The anti-cancer activity of rapamycin is increased in combination with acetazolamide

**Figure 4:**
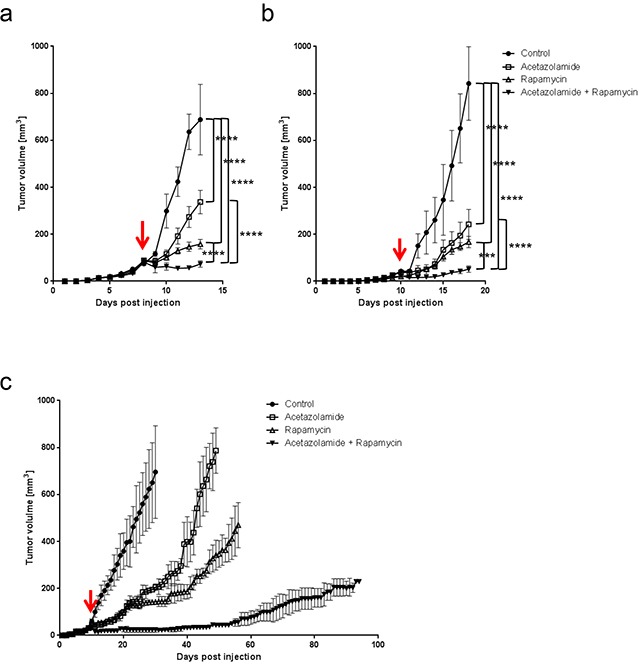
Acetazolamide potentiates the anti-cancer efficacy of rapamycin **A.** HT29 xenograft growth curves for treatments with vehicle, acetazolamide (40 mg/kg daily), rapamycin (3 mg/kg daily) or a combination of both. **B.** MC-38 allograft growth curves with treatments as under a. **C.** Long term effect of acetazolamide/rapamycin treatments on the growth of HT29 xenografts. Arrows denote the start of treatment at 25 mm^3^ graft volume. **** p<0.0001, *** p<0.001, n=5/group, Two-way ANOVA.

**Figure 5:**
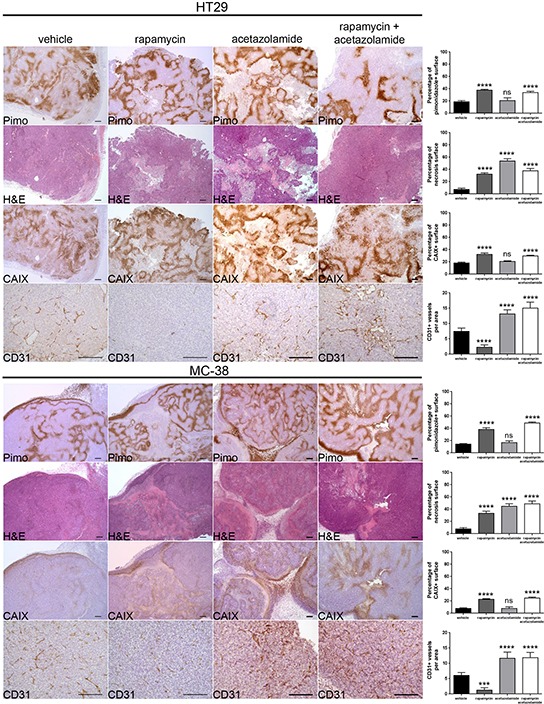
Acetazolamide increases necrosis and tumor vasculature Percentage of tumor hypoxia (pimonidazole positive surface), tumor necrosis (light pink stained surface in H&E) and CAIX expression (CAIX positive surface) were compared for vehicle, rapamycin, acetazolamide and combined treatment of HT29 xenograft and MC-38 allograft in 10 representative sections of 3368 × 2668 μm for three different tumors. Tumor vasculature was analyzed by counting CD31 positive vessels in 10 representative sections of 200 × 200 μm for three different tumors. Scale bars, 200 μm. Bar charts represent mean, error bars represent SD. **** p<0.0001, *** p<0.001, ns=not significant, One-way ANOVA.

**Figure 6:**
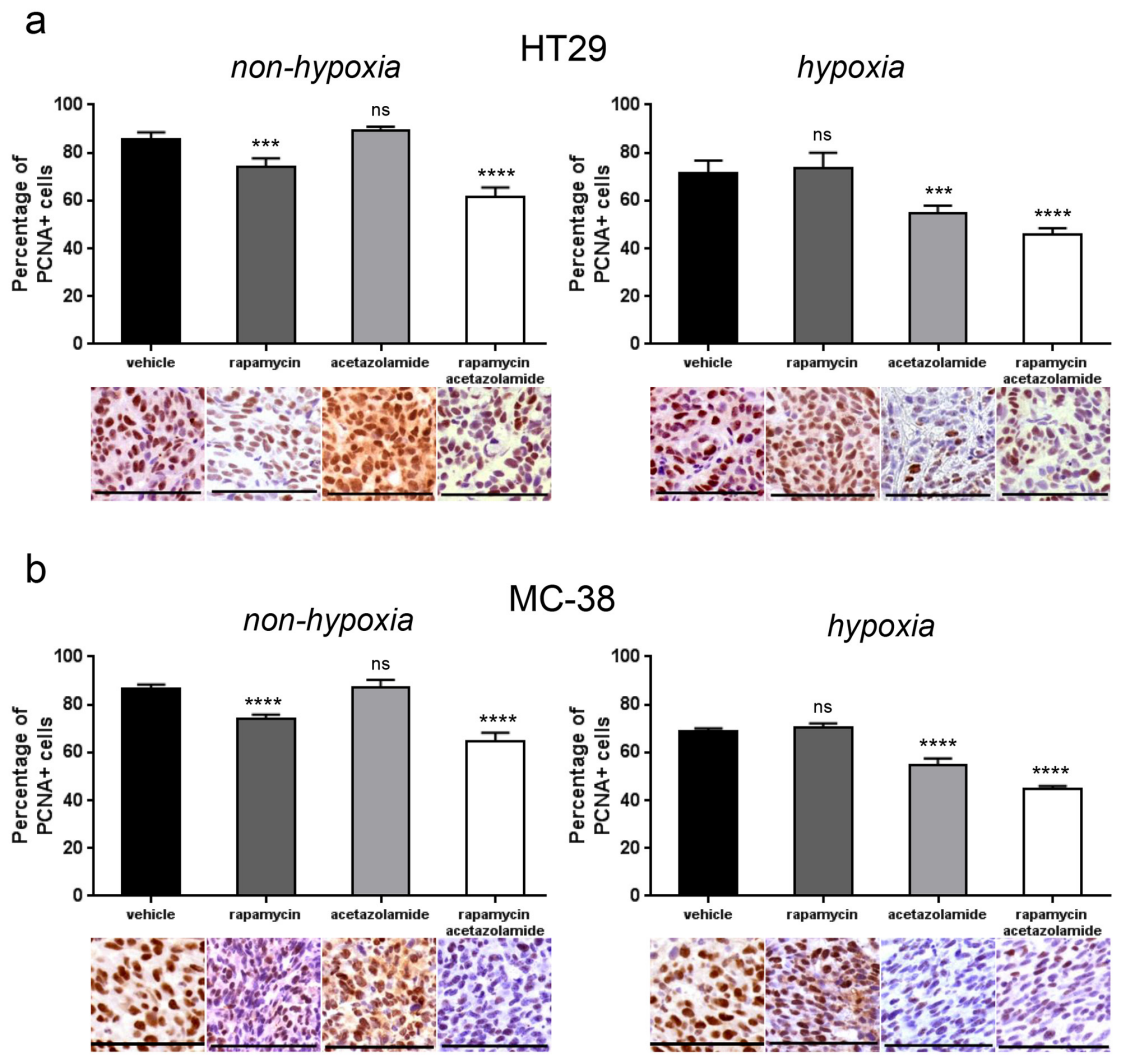
CAIX inhibition by acetazolamide has an anti-proliferative effect on hypoxic tumor regions **A. B.** Percentage of PCNA positive cancer cells was counted in 10 representative pimonidazole positive and pimonidazole negative zones of a 100 × 100 μm surface for three different HT29 (A) and MC-38 (B) tumors respectively. Bar charts represent mean, error bars represent SD. **** p<0.0001, *** p<0.001, ns=not significant, Student's t-test. Representative image section below corresponding bar chart, scale bar, 100 μm.

Since rapamycin treatment increases the expression of CAIX, and CAIX is known to promote cancer progression, we next asked whether blocking CAIX activity would improve the efficacy of rapamycin. To test this, we treated nude mice bearing HT29 tumor xenografts or C57BL/6 mice bearing MC-38 allografts with acetazolamide alone or in combination with rapamycin. Although acetazolamide is a non-specific inhibitor of carbonic anhydrase enzymes, we opted for it as it is a regularly used agent and well tolerated by patients [20]. We found that both acetazolamide and rapamycin alone reduced tumor growth. The effect was however significantly stronger when both agents were combined (Figure 4A and 4B). The effect was long-lasting as, after three months of treatment, HT29 tumor xenografts did still not exceed a size of 230 mm^3^ (Figure 4C). Histological analysis revealed that acetazolamide increased tumor necrosis (from 7.4 % to 53.8 % and from 7.8 % to 44.8 % in HT29 and MC-38 tumors respectively) and the number of tumor blood vessels (increase by 78.4 % in HT29 and 93.3 % in MC-38 tumors) (Figure 5). Interestingly, acetazolamide reduced proliferation in hypoxic (from 71.4 % to 54.6 % and from 68.9 % to 54.5 % in HT29 and MC-38 tumors respectively) but not in non-hypoxic regions, whereas the opposite was observed with rapamycin. Combined treatment with rapamycin and acetazolamide produced antiproliferative effects in both the hypoxic and non-hypoxic area (Figure 6A and 6B). Taken together, these data demonstrate that combining acetazolamide with rapamycin exhibits stronger anti-cancer effects than either drug alone.

### Targeting CAIX potentiates the anti-cancer efficacy of rapamycin

**Figure 7:**
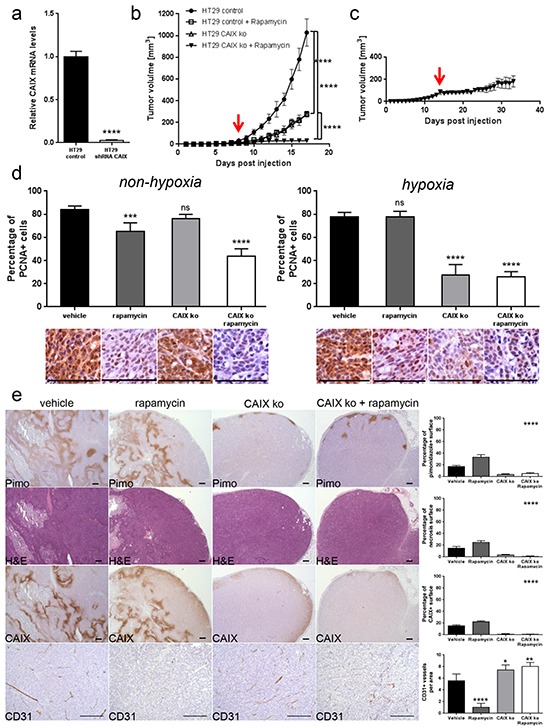
CAIX knockdown exhibits an anti-proliferative effect on hypoxic tumor regions and potentiates anti-cancer efficacy of rapamycin **A.** CAIX knockdown in HT29 cells was confirmed by qRT-PCR. **** p<0.0001, Student's t-test. **B.** HT29 control and HT29 shCAIX xenograft growth curves for treatments with vehicle or rapamycin (3 mg/kg daily). **C.** Long term effect of rapamycin treatment in HT29 shCAIX xenografts. Arrows denote the start of rapamycin treatment at 25 mm^3^ graft volume. **** p<0.0001, n=5/group, Two-way ANOVA. **D.** Percentage of PCNA positive cancer cells was counted in 10 representative pimonidazole positive and pimonidazole negative zones of a 100 × 100 μm surface for three different HT29 control or HT29 shCAIX tumors. Bar charts represent mean, error bars represent SD. **** p<0.0001, *** p<0.001, ns=not significant, Student's t-test. Representative image section below corresponding bar chart, scale bar, 100 μm. E. Percentage of tumor hypoxia (pimonidazole positive surface), tumor necrosis (light pink stained surface in H&E) and CAIX expression (CAIX positive surface) were compared for control and CAIX knockdown HT29 xenograft with vehicle or rapamycin treatment in 10 representative sections of 3368 × 2668 μm for three different tumors. Tumor vasculature was analyzed by counting CD31 positive vessels in 10 representative sections of 200 × 200 μm for three different tumors. Scale bars, 200 μm. Bar charts represent mean, error bars represent SD. **** p<0.0001, ** p<0.01, * p<0.05, One-way ANOVA.

We next investigated whether among the different carbonic anhydrase enzymes, targeting specifically CAIX would potentiate the anti-cancer effect of rapamycin. To test this, we knocked down CAIX in HT29 cells (HT29 shCAIX) by infecting HT29 cells with lentiviruses containing CAIX shRNA. Knockdown of CAIX was confirmed by qRT-PCR (Figure 7A) and by immunostaining of tumor xenografts (Figure 7E). HT29 shCAIX or control HT29 expressing a scramble shRNA were injected subcutaneously into nude mice, and tumor growth was monitored. We found that the growth of HT29 shCAIX tumor xenografts was reduced compared to control HT29 (Figure 7B). Rapamycin treatment further reduced the growth of HT29 shCAIX tumor xenografts. This growth reduction was also stronger compared to rapamycin treated control xenografts. The tumor size of shCAIX xenografts was still below 200 mm^3^ after a long term rapamycin treatment of 20 days (Figure 7c). CAIX knockdown reduced proliferation in the hypoxic but not the non-hypoxic tumor regions. A combined inhibition of mTORC1 and CAIX provoked antiproliferative effects in both the hypoxic and non-hypoxic area (Figure 7d). Furthermore, CAIX knockdown decreased tumor hypoxia and tumor necrosis, but increased CD31 positive tumor vasculature (Figure 7e). Taken together, these results emphasize that an inhibition of CAIX potentiates the efficacy of rapamycin.

## DISCUSSION

Targeting mTOR is a promising approach in cancer therapy. However, in the course of time, cancers adapt to mTOR inhibition and develop resistance mechanisms that are responsible for the limited efficacy of mTOR inhibitors [2]. To date, most identified resistance mechanisms are the consequence of an abrogation of negative feedback loops following mTORC1 inhibition [21]. In this study, we show that the hypoxic microenvironment of a tumor is resistant to mTOR inhibitors, and we propose that targeting the hypoxic region of a tumor is an effective approach to enhance their anti-tumor efficacy.

Most tumors are characterized by regions of hypoxia that contribute to chemo- and radioresistance [9]. Our findings show that hypoxia also contributes to resistance to mTOR inhibitors. Indeed, we outline that mTORC1 activity is not present in hypoxic regions of a tumor as evidenced by a mutually exclusive staining pattern of pimonidazole and pS6 (Figure 1A). Consistent with this observation, it was reported that mTORC1 activity negatively correlated with HIF-1α expression in renal cancer xenografts [22]. Furthermore, we observe that rapamycin reduces cancer cell proliferation in non-hypoxic but not in hypoxic regions of a tumor (Figure 2A). This further supports our hypothesis that hypoxic cancer cells proliferate independently of mTORC1. Consistent with our observation that rapamycin exerts a tumor region selective anti-proliferative effect, it was reported that, whereas rapamycin decreases proliferation in the outer well vascularized part of tumors, it promotes cancer cell proliferation in hypovacular areas of tumors [23].

Another explanation for the loss of antiproliferative activity of rapamycin in hypoxic tumor regions is a reduced access of rapamycin to hypoxic areas. Indeed, rapamycin is well characterized for its anti-angiogenic properties [24, 25]. Consistent with this, we found that rapamycin decreased the number of blood vessels and increased tumor hypoxia which could secondarily lead to reduced rapamycin delivery to hypoxic tumor regions.

Adaptation of cancer cells to hypoxia involves the expression of many genes that are under the control of the transcription factor HIF-1 [9, 26]. Interestingly, several studies have demonstrated that HIF-1 expression is positively regulated by mTORC1, which would argue for a control of hypoxic gene activation by mTORC1 [27–29]. The role of mTORC1 in regulating hypoxia-induced HIF-1 expression seems however complex and might depend on the level of hypoxia. Actually, whereas rapalogs were shown to partially reduce the level of HIF-1 induced by mild hypoxia (3 % O_2_), this effect was abrogated in conditions of severe hypoxia (0.3 % O_2_) [22]. Consistent with this, we demonstrate that rapamycin increases the expression of CAIX which is under the control of HIF-1 (Figure 3). This further suggests that HIF-1-mediated hypoxic gene activation is not controlled by mTORC1 in the hypoxic tumor microenvironment.

Among the various proteins that are controlled by HIF-1, mounting evidence has outlined the importance of CAIX in cancer biology. In different experimental settings, CAIX knockdown reduced the growth rate of tumor xenografts, and conversely, overexpression of CAIX increased tumor growth [13–15]. Similarly, we see that CAIX knockdown slows the progression of tumor xenografts (Figure 7B and 7C). We further show that cancer cell proliferation in hypoxic zones is reduced following knockdown of CAIX (Figure 7D), highlighting the importance of CAIX to maintain favorable growth conditions in a hypoxic environment. Similarly, *in vitro*, it was demonstrated that CAIX expression was associated with proliferation in hypoxic areas of cancer cell spheroids [14]. Therefore, CAIX actively participates in tumor growth, and accordingly, selective inhibitors of CAIX are currently tested in clinical trials [30, 31].

The primary function of CAIX is the hydration of carbon dioxide to bicarbonate and proton, maintaining an alkaline intracellular pH and promoting an acidic extracellular space [30, 32]. Our observation therefore suggests that increasing the tumor pH via inhibition of CAIX might potentiate the efficacy of mTOR inhibitors. Of note, acidic extracellular pH inhibits mTORC1 function [33, 34]. Consequently, in addition to hypoxia, acidic tumor pH might further downregulate mTORC1 activity and promote an mTORC1-independent cancer cell proliferation. Besides CAIX, several other proteins are implicated in the regulation of tumor pH, including bicarbonate transporters, proton pump, Na^+^/H^+^ exchanger 1 or monocarboxylate transporter [35, 36]. Future studies will characterize whether modifying the functions of these proteins might influence the anti-cancer efficacy of mTORC1 inhibitors.

A combination of rapamycin with CAIX inhibitors is further justified by the observation that rapamycin treatment increases the extent of hypoxia in tumors which is associated with an increased expression of CAIX (Figure 3). This observation relies mostly on the reduction of the number of tumor blood vessels induced by rapamycin. Therefore, targeting CAIX is of importance in combination with treatments that increase tumor hypoxia such as anti-angiogenesis therapies. A reinforcement of the anti-tumoral activity of the anti-VEGF antibody bevacizumab by an inhibition of CAIX further supports this hypothesis [14].

Our study further underlines the benefit of therapeutic approaches that target the hypoxic tumor response in combination with anti-angiogenic agents. Consistent with our findings, such an approach has shown promising results in other pre-clinical models [37]. For example, targeting HIF1α increases the efficacy of bevacizumab in neuroblastoma xenografts [38]. A similar effect has also been reported when bevacizumab is combined with acetazolamide [14] [39]. More importantly, such strategies are also tested in clinical trials [37].

Combining inhibitors of the hypoxic tumor response with rapamycin could be particularly beneficial compared to their combinations with other anti-angiogenic drugs. Indeed the importance of mTORC1 in tumors is not restricted to tumor vessels; mTORC1 also affects cancer cells and cells present in the tumor microenvironment. Hence, blocking mTORC1 can lead to unexpected effects due to the variety and complexity of cellular responses induced by mTORC1 inhibition. For example it was demonstrated that in a mouse model of K-Ras-induced pancreatic tumors, mTORC1 has opposing effect on tumor cell proliferation in nutrient-rich versus nutrient-depleted conditions. Whereas it blocks tumor cell proliferation in vascular tumor areas, it increases tumor cell proliferation in hypovascular, nutrient depleted regions, resulting overall in tumor growth [23]. This further outlines that targeting the hypoxic tumor regions can potentiate the efficacy of mTORC1 inhibitors.

Preclinical studies have underlined a role of acetazolamide in restraining cellular processes involved in tumor progression. For instance, renal cancer cell invasiveness and survival is diminished by acetazolamide *in vitro* [40, 41]. In addition, acetazolamide has shown anti-tumor properties in murine models [14, 42, 43]. Our data further demonstrate that acetazolamide-induced tumor growth inhibition is associated with reduced cancer cell proliferation in hypoxic zones (Figure 6A and 6B), which is in accordance with what we observe following selective knockdown of CAIX (Figure 7D). However, whereas CAIX knockdown significantly reduces tumor necrosis (Figure 7E), treatment with acetazolamide results in increased necrosis (Figure 5). The non-selective property of acetazolamide for the inhibition of carbonic anhydrase isoforms might explain this discrepancy.

We found that acetazolamide treatment or CAIX knockdown in cancer cells significantly increased the number of blood vessels in tumors. The molecular mechanisms underlying this effect remain however uncharacterized. Acidity is known to reduce endothelial cell proliferation and migration as well as endothelial cell sprouting [44, 45]. Hence, increasing intratumoral pH following the inhibition of carbonic anhydrase enzymes could favor tumor angiogenesis. A direct effect of acetazolamide on endothelial cells has also to be considered. Indeed, carbonic anhydrase II has been detected on tumor endothelial cells, and its inhibition could affect endothelial cell functions that are relevant to angiogenesis [46]. Clearly, additional investigations are needed to fully characterize the effect of targeting carbonic anhydrase enzymes on tumor endothelium.

The mechanisms responsible for the anti-cancer activity of rapamycin are complex and not restricted to its effect on tumor and endothelial cells [2]. Consistent with this, the antiproliferative effect of rapamycin on cancer cells that we describe here is small and cannot solely account for the anti-cancer activity of rapamycin in our study as well as in patients. Recent studies have demonstrated the complex role of mTORC1 in the immune system [47]. Although classically rapamycin is used as an immunosuppressive drug, several evidence point out that in distinct conditions rapamycin favors CD8^+^ memory response and hence mediates an immunostimulatory response [48–50]. Thus, besides the antiproliferative effects of rapamycin, several other aspects contribute to the anti-cancer activity of rapamycin and need to be fully identified. Our experimental set-up did not allow to investigate the tumor immune response. Hence future experiments will determine whether inhibition of the hypoxic tumor response in combination with rapamycin positively influences tumor immune response.

Tumors are characterized by genetic and epigenetic alterations that play a fundamental role in malignant transformation. However, genetic and epigenetic alterations display a spatial and temporal heterogeneity and vary in consequence of anti-cancer treatments [51]. This tumor heterogeneity may account for the limited efficacy of targeting therapies. Our findings further emphasize that the tumor microenvironment affects signaling pathways that participate in tumor growth, adding another level of complexity to the molecular heterogeneity of tumors.

In summary, our study shows that mTORC1 activity is reduced in hypoxic tumor regions which consequently resist to mTORC1 inhibitors. Targeting the hypoxic microenvironment represents a novel therapeutic strategy to potentiate the efficacy of mTORC1 inhibitors.

## MATERIALS AND METHODS

### Cell culture, reagents, antibodies

Human colorectal adenocarcinoma cell line HT29 were purchased from American Type Culture Collection and murine colon adenocarcinoma cell line MC-38 were kindly provided by Dr. Jeffrey Schlom (National Cancer Institute, NIH) [52]. Cell lines were cultured in Dulbecco's Modified Eagle's Medium - high glucose (DMEM) (Sigma-Aldrich) supplemented with 10 % FBS and 1 % streptomycin/penicillin. Rapamycin was from LC Laboratories. Acetazolamide was from Sigma-Aldrich. For cell culture, rapamycin was dissolved in dimethyl sulfoxide (DMSO). For mouse models, rapamycin and acetazolamide were dissolved in DMSO at the below-mentioned dosage and diluted 1:5 in PBS-Tween-PEG (89.6 % phosphate-buffered saline (PBS), 5.2 % Tween 20, 5.2 % poly(ethylene glycol)). Solid pimonidazole HCl as part of the Hydroxyprobe^TM^-1 Kit was from hpi Hydroxyprobe Inc. For immunohistochemical staining, the following primary antibodies and concentrations were used: Anti-phospho S6 ribosomal protein antibody (1:100) (M3500; Spring Bioscience Corp.), anti-CD31/PECAM-1 antibody (1:50) (RB-10333; ThermoFisher Scientific), anti-carbonic anhydrase IX antibody (1:100) (NB100-417; Novus Biologicals), anti-PCNA antibody (1:50) (ab2426; Abcam) and anti-phospho-4E-BP1 antibody (1:100) (2855; Cell Signaling Technology).

### Proliferation assay

HT29 cells were plated on 96 well plates (Costar) at 5′000 cells per well, cultured in DMEM in hypoxia O_2_ 1 % or non-hypoxia O_2_ 21 % and treated with DMSO or 100 nM rapamycin for 48 and 96 hours. Cellular proliferation was monitored after 48 and 96 hours with CellTiter 96 AQ_ueous_ One Solution Cell Proliferation Assay (MTS) (Promega Corporation) by following the manufacturer's instructions. Absorbance at 492 nm was measured 30 minutes after compound administration. Experiment was performed in quadruplicates and repeated three times.

### Stable transfection

To knockdown CAIX in HT29 cells, we used lentivirus produced by the lenti-vpak packaging kit from OriGene following the manufacturer's instructions and containing the CAIX gene-specific shRNA expression vector (TL314250B) or a negative control non-effective HuSH 29-mer scrambled shRNA cassette (TR30021) in pGFP-C-shLenti plasmid. Cells were grown under selective pressure (puromycin 10 μg/ml), and the knockdown efficiency was tested by qRT-PCR.

### qRT-PCR

RNA extraction was performed using RNeasy Mini Kit from Qiagen by following the manufacturer's instructions. We used 500 ng of RNA for reverse transcription with SuperScript II Reverse Transcriptase from ThermoFisher Scientific. The resulting cDNA was used for qRT-PCR (Rotor-Gene Q from Qiagen). qRT-PCR were set up in triplicates with KAPA SYBR FAST qPCR Kit Master Mix Universal KK4602 from Kapa Biosystems. Relative gene expression and fold changes were determined using the 2^−ΔΔ*CT*^ method using GAPDH as an internal control [53]. Primer sequences were: human CAIX forward GGG TGT CAT CTG GAC TGT GTT, human CAIX reverse CTT CTG TGC TGC CTT CTC ATC, human GAPDH forward CCA TGG GGA AGG TGA AGG TC, human GAPDH reverse ACG TAC TCA GCG CCA GCA TC, mouse CAIX forward GCT GTC CCA TTT GGA AGA AA, mouse CAIX reverse GGA AGG AAG CCT CAA TCG TT, mouse GAPDH forward AAG AGG GAT GCT GCC CTT A, mouse GAPDH reverse TTG TCT ACG GGA CGA GGA AA.

### Immunohistochemistry

Xeno- and allografts were fixed in formaline 4 % overnight, dehydrated with ethanol and paraffin-embedded. Sections of 3 μm were obtained using MICROM HS355S microtome, and tissue sections were mounted on Superfrost Plus slides. Slides were then deparaffinized and rehydrated with xylol and alcohol. After antigen retrieval (citrate pH 6.0 or TRIS/EDTA pH 9.0), sections were immunostained using above-mentioned primary antibodies for 60 minutes incubation time and subsequently incubated with Dako EnVision HRP secondary rabbit or mouse antibody for 30 minutes. One section from each xenograft and allograft tumor and three tumors for each condition were analyzed for each staining. Carl Zeiss Axioscope, AxioCam MRc and AxioVision 40V 4.6.3.0 software from Carl Zeiss Imaging Solutions GmbH were used for microscopy, imaging acquisition and image processing. Histology analysis was performed by two researchers blinded to groupings. Percentage of tumor hypoxia (pimonidazole positive surface), tumor necrosis (light pink stained surface in H&E) and CAIX expression (CAIX positive surface) were measured quantitatively using ImageJ 1.46r Threshold Colour Plugin analysis by analyzing 10 representative images of 3368 × 2668 μm for each condition in three different tumors. Tumor vasculature was analyzed by counting CD31 positive vessels in 10 representative sections of 200 × 200 μm for three different tumors. PCNA positive and PCNA negative cancer cells were counted in 10 representative pimonidazole positive and pimonidazole negative vital tumor zones of a 100 × 100 μm surface for three different HT29 and MC-38 tumors. Proliferation rate was calculated by dividing the number of PCNA positive cancer cells by the number of PCNA positive and PCNA negative cancer cells.

### Mouse models

Animal experiments were in accordance with the Swiss federal animal regulations and approved by the local veterinary office. Female nude and C57BL/6 eight-week old mice were purchased from Janvier Labs. HT29 cells (3 × 10^6^) and MC-38 (1 × 10^6^) cells were injected subcutaneously into the right flank. Once the tumor xeno-/allografts reached a mean size of 25 mm^3^, mice were randomized into different groups (n=5/group; groups “vehicle” – “acetazolamide” – “rapamycin” – “acetazolamide and rapamycin”) and treated daily with rapamycin (3 mg/kg/day, intraperitoneally), acetazolamide (40 mg/kg/day, intraperitoneally), a combination of both or vehicle only. Tumor volumes were measured daily using a caliper and calculated with the formula V = A * B * C * π / 6 where A is the length, B the width and C the height of the tumor. Animals were sacrificed once the biggest tumor of vehicle treated mice reached the size of 1000 mm^3^ (defined as interruption criterion according to veterinary recommendations). Pimonidazole HCl 15 mg/ml in NaCl 0.9 % was injected intraperitoneally at a dosage of 60 mg/kg, 60 minutes before tissue harvest. Tumors were excised and samples processed for immunohistochemical analysis.

### Statistics

Statistical analysis including Student's t-test, One-way ANOVA and Two-way ANOVA were carried out as appropriate using GraphPad Prism version 6.05.

## SUPPLEMENTARY FIGURE



## References

[1] Guertin DA, Sabatini DM (2007). Defining the role of mTOR in cancer. Cancer Cell.

[2] Dufour M, Dormond-Meuwly A, Demartines N, Dormond O (2011). Targeting the Mammalian Target of Rapamycin (mTOR) in Cancer Therapy: Lessons from Past and Future Perspectives. Cancers (Basel).

[3] Porta C, Paglino C, Mosca A (2014). Targeting PI3K/Akt/mTOR Signaling in Cancer. Front Oncol.

[4] Chandarlapaty S (2012). Negative feedback and adaptive resistance to the targeted therapy of cancer. Cancer Discov.

[5] Laplante M, Sabatini DM (2012). mTOR signaling in growth control and disease. Cell.

[6] Shimobayashi M, Hall MN (2014). Making new contacts: the mTOR network in metabolism and signalling crosstalk. Nat Rev Mol Cell Biol.

[7] Rodon J, Dienstmann R, Serra V, Tabernero J (2013). Development of PI3K inhibitors: lessons learned from early clinical trials. Nat Rev Clin Oncol.

[8] Grabiner BC, Nardi V, Birsoy K, Possemato R, Shen K, Sinha S, Jordan A, Beck AH, Sabatini DM (2014). A diverse array of cancer-associated MTOR mutations are hyperactivating and can predict rapamycin sensitivity. Cancer Discov.

[9] Wilson WR, Hay MP (2011). Targeting hypoxia in cancer therapy. Nat Rev Cancer.

[10] Chiche J, Brahimi-Horn MC, Pouyssegur J (2010). Tumour hypoxia induces a metabolic shift causing acidosis: a common feature in cancer. J Cell Mol Med.

[11] Keith B, Johnson RS, Simon MC (2012). HIF1alpha and HIF2alpha: sibling rivalry in hypoxic tumour growth and progression. Nat Rev Cancer.

[12] Sedlakova O, Svastova E, Takacova M, Kopacek J, Pastorek J, Pastorekova S (2014). Carbonic anhydrase IX, a hypoxia-induced catalytic component of the pH regulating machinery in tumors. Front Physiol.

[13] Chiche J, Ilc K, Laferriere J, Trottier E, Dayan F, Mazure NM, Brahimi-Horn MC, Pouyssegur J (2009). Hypoxia-inducible carbonic anhydrase IX and XII promote tumor cell growth by counteracting acidosis through the regulation of the intracellular pH. Cancer research.

[14] McIntyre A, Patiar S, Wigfield S, Li JL, Ledaki I, Turley H, Leek R, Snell C, Gatter K, Sly WS, Vaughan-Jones RD, Swietach P, Harris AL (2012). Carbonic anhydrase IX promotes tumor growth and necrosis in vivo and inhibition enhances anti-VEGF therapy. Clinical cancer research.

[15] Lou Y, McDonald PC, Oloumi A, Chia S, Ostlund C, Ahmadi A, Kyle A, Auf dem Keller U, Leung S, Huntsman D, Clarke B, Sutherland BW, Waterhouse D, Bally M, Roskelley C, Overall CM (2011). Targeting tumor hypoxia: suppression of breast tumor growth and metastasis by novel carbonic anhydrase IX inhibitors. Cancer research.

[16] Dubois L, Peeters S, Lieuwes NG, Geusens N, Thiry A, Wigfield S, Carta F, McIntyre A, Scozzafava A, Dogne JM, Supuran CT, Harris AL, Masereel B, Lambin P (2011). Specific inhibition of carbonic anhydrase IX activity enhances the in vivo therapeutic effect of tumor irradiation. Radiother Oncol.

[17] Beasley NJ, Wykoff CC, Watson PH, Leek R, Turley H, Gatter K, Pastorek J, Cox GJ, Ratcliffe P, Harris AL (2001). Carbonic anhydrase IX, an endogenous hypoxia marker, expression in head and neck squamous cell carcinoma and its relationship to hypoxia, necrosis, and microvessel density. Cancer research.

[18] Wykoff CC, Beasley NJ, Watson PH, Turner KJ, Pastorek J, Sibtain A, Wilson GD, Turley H, Talks KL, Maxwell PH, Pugh CW, Ratcliffe PJ, Harris AL (2000). Hypoxia-inducible expression of tumor-associated carbonic anhydrases. Cancer research.

[19] DeYoung MP, Horak P, Sofer A, Sgroi D, Ellisen LW (2008). Hypoxia regulates TSC1/2-mTOR signaling and tumor suppression through REDD1-mediated 14-3-3 shuttling. Genes Dev.

[20] Kassamali R, Sica DA (2011). Acetazolamide: a forgotten diuretic agent. Cardiol Rev.

[21] Efeyan A, Sabatini DM (2010). mTOR and cancer: many loops in one pathway. Curr Opin Cell Biol.

[22] Knaup KX, Jozefowski K, Schmidt R, Bernhardt WM, Weidemann A, Juergensen JS, Warnecke C, Eckardt KU, Wiesener MS (2009). Mutual regulation of hypoxia-inducible factor and mammalian target of rapamycin as a function of oxygen availability. Mol Cancer Res.

[23] Palm W, Park Y, Wright K, Pavlova NN, Tuveson DA, Thompson CB (2015). The Utilization of Extracellular Proteins as Nutrients Is Suppressed by mTORC1. Cell.

[24] Guba M, von Breitenbuch P, Steinbauer M, Koehl G, Flegel S, Hornung M, Bruns CJ, Zuelke C, Farkas S, Anthuber M, Jauch KW, Geissler EK (2002). Rapamycin inhibits primary and metastatic tumor growth by antiangiogenesis: involvement of vascular endothelial growth factor. Nature medicine.

[25] Vinals F, Chambard JC, Pouyssegur J (1999). p70 S6 kinase-mediated protein synthesis is a critical step for vascular endothelial cell proliferation. The Journal of biological chemistry.

[26] Semenza GL (2012). Hypoxia-inducible factors: mediators of cancer progression and targets for cancer therapy. Trends Pharmacol Sci.

[27] Zhong H, Chiles K, Feldser D, Laughner E, Hanrahan C, Georgescu MM, Simons JW, Semenza GL (2000). Modulation of hypoxia-inducible factor 1alpha expression by the epidermal growth factor/phosphatidylinositol 3-kinase/PTEN/AKT/FRAP pathway in human prostate cancer cells: implications for tumor angiogenesis and therapeutics. Cancer research.

[28] Hudson CC, Liu M, Chiang GG, Otterness DM, Loomis DC, Kaper F, Giaccia AJ, Abraham RT (2002). Regulation of hypoxia-inducible factor 1alpha expression and function by the mammalian target of rapamycin. Mol Cell Biol.

[29] Thomas GV, Tran C, Mellinghoff IK, Welsbie DS, Chan E, Fueger B, Czernin J, Sawyers CL (2006). Hypoxia-inducible factor determines sensitivity to inhibitors of mTOR in kidney cancer. Nature medicine.

[30] McDonald PC, Winum JY, Supuran CT, Dedhar S (2012). Recent developments in targeting carbonic anhydrase IX for cancer therapeutics. Oncotarget.

[31] Supuran CT (2008). Carbonic anhydrases: novel therapeutic applications for inhibitors and activators. Nat Rev Drug Discov.

[32] Swietach P, Patiar S, Supuran CT, Harris AL, Vaughan-Jones RD (2009). The role of carbonic anhydrase 9 in regulating extracellular and intracellular ph in three-dimensional tumor cell growths. The Journal of biological chemistry.

[33] Pouyssegur J, Chambard JC, Franchi A, Paris S, Van Obberghen-Schilling E (1982). Growth factor activation of an amiloride-sensitive Na+/H+ exchange system in quiescent fibroblasts: coupling to ribosomal protein S6 phosphorylation. Proceedings of the National Academy of Sciences of the United States of America.

[34] Balgi AD, Diering GH, Donohue E, Lam KK, Fonseca BD, Zimmerman C, Numata M, Roberge M (2011). Regulation of mTORC1 signaling by pH. PLoS One.

[35] Pouyssegur J, Dayan F, Mazure NM (2006). Hypoxia signalling in cancer and approaches to enforce tumour regression. Nature.

[36] Neri D, Supuran CT (2011). Interfering with pH regulation in tumours as a therapeutic strategy. Nat Rev Drug Discov.

[37] McIntyre A, Harris AL (2015). Metabolic and hypoxic adaptation to anti-angiogenic therapy: a target for induced essentiality. EMBO molecular medicine.

[38] Hartwich J, Orr WS, Ng CY, Spence Y, Morton C, Davidoff AM (2013). HIF-1alpha activation mediates resistance to anti-angiogenic therapy in neuroblastoma xenografts. Journal of pediatric surgery.

[39] Vaeteewoottacharn K, Kariya R, Dana P, Fujikawa S, Matsuda K, Ohkuma K, Kudo E, Kraiklang R, Wongkham C, Wongkham S, Okada S (2016). Inhibition of carbonic anhydrase potentiates bevacizumab treatment in cholangiocarcinoma. Tumour biology.

[40] Parkkila S, Rajaniemi H, Parkkila AK, Kivela J, Waheed A, Pastorekova S, Pastorek J, Sly WS (2000). Carbonic anhydrase inhibitor suppresses invasion of renal cancer cells in vitro. Proceedings of the National Academy of Sciences of the United States of America.

[41] Cianchi F, Vinci MC, Supuran CT, Peruzzi B, De Giuli P, Fasolis G, Perigli G, Pastorekova S, Papucci L, Pini A, Masini E, Puccetti L (2010). Selective inhibition of carbonic anhydrase IX decreases cell proliferation and induces ceramide-mediated apoptosis in human cancer cells. The Journal of pharmacology and experimental therapeutics.

[42] Teicher BA, Liu SD, Liu JT, Holden SA, Herman TS (1993). A carbonic anhydrase inhibitor as a potential modulator of cancer therapies. Anticancer research.

[43] Mokhtari RB, Kumar S, Islam SS, Yazdanpanah M, Adeli K, Cutz E, Yeger H (2013). Combination of carbonic anhydrase inhibitor, acetazolamide, and sulforaphane, reduces the viability and growth of bronchial carcinoid cell lines. BMC cancer.

[44] D'Arcangelo D, Facchiano F, Barlucchi LM, Melillo G, Illi B, Testolin L, Gaetano C, Capogrossi MC (2000). Acidosis inhibits endothelial cell apoptosis and function and induces basic fibroblast growth factor and vascular endothelial growth factor expression. Circulation research.

[45] Burbridge MF, West DC, Atassi G, Tucker GC (1999). The effect of extracellular pH on angiogenesis in vitro. Angiogenesis.

[46] Yoshiura K, Nakaoka T, Nishishita T, Sato K, Yamamoto A, Shimada S, Saida T, Kawakami Y, Takahashi TA, Fukuda H, Imajoh-Ohmi S, Oyaizu N, Yamashita N (2005). Carbonic anhydrase II is a tumor vessel endothelium-associated antigen targeted by dendritic cell therapy. Clinical cancer research.

[47] Chi H (2012). Regulation and function of mTOR signalling in T cell fate decisions. Nature reviews Immunology.

[48] Araki K, Turner AP, Shaffer VO, Gangappa S, Keller SA, Bachmann MF, Larsen CP, Ahmed R (2009). mTOR regulates memory CD8 T-cell differentiation. Nature.

[49] Scholz G, Jandus C, Zhang L, Grandclement C, Lopez-Mejia IC, Soneson C, Delorenzi M, Fajas L, Held W, Dormond O, Romero P (2016). Modulation of mTOR Signalling Triggers the Formation of Stem Cell-like Memory T Cells. EBioMedicine.

[50] Li Q, Rao R, Vazzana J, Goedegebuure P, Odunsi K, Gillanders W, Shrikant PA (2012). Regulating mammalian target of rapamycin to tune vaccination-induced CD8(+) T cell responses for tumor immunity. Journal of immunology.

[51] Kleppe M, Levine RL (2014). Tumor heterogeneity confounds and illuminates: assessing the implications. Nature medicine.

[52] Robbins PF, Kantor JA, Salgaller M, Hand PH, Fernsten PD, Schlom J (1991). Transduction and expression of the human carcinoembryonic antigen gene in a murine colon carcinoma cell line. Cancer research.

[53] Schmittgen TD, Livak KJ (2008). Analyzing real-time PCR data by the comparative C(T) method. Nat Protoc.

