# Supplementary material for: Targeting carbonic anhydrase IX improves the anti-cancer efficacy of mTOR inhibitors
Source: Oncotarget. 2016 May 2;7(24):36666–80. doi: 10.18632/oncotarget.9134 (PMC5095030; doi:10.18632/oncotarget.9134)
Supplement: Supplementary file 1 [file oncotarget-07-36666-s001.pdf]

SUPPLEMENTARY FIGURE

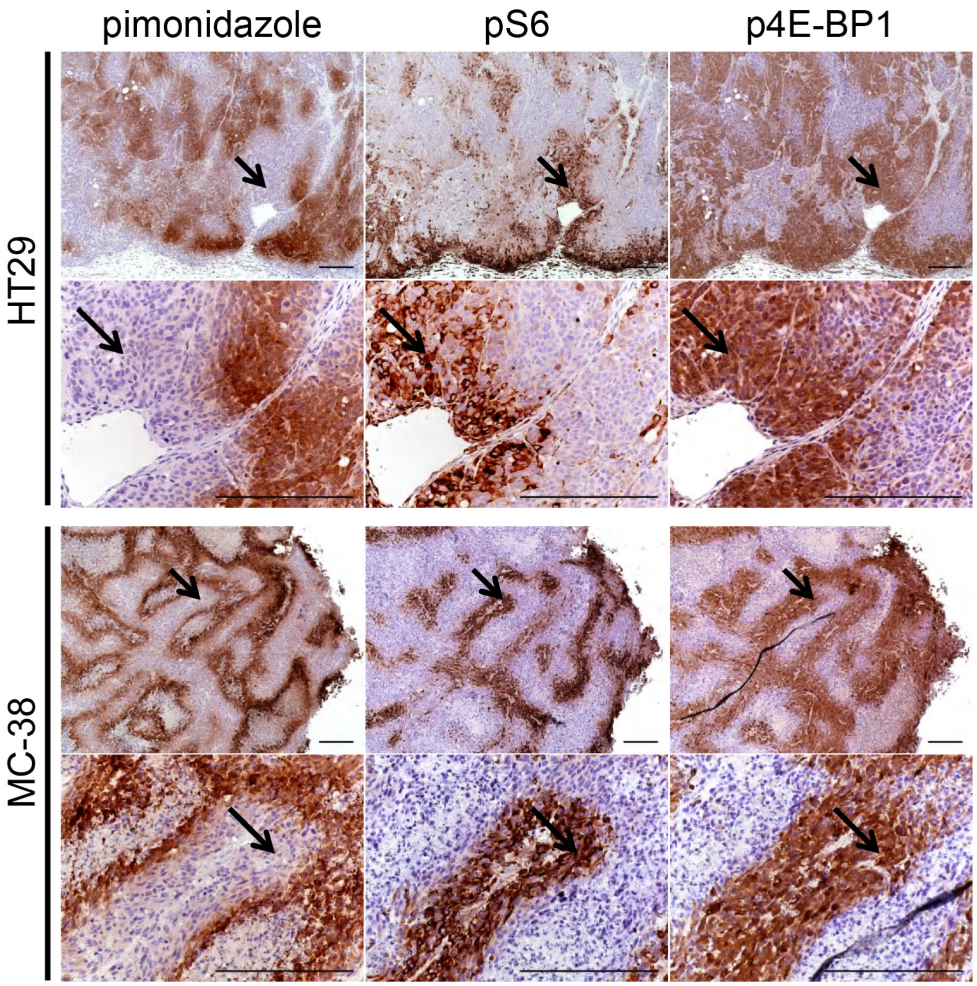

Supplementary Figure S1: mTORC1 activity is absent in hypoxic regions of a tumor. serial sections of HT29 tumor xenografts and MC-38 tumor allografts were stained for pimonidazole, pS6 or PCNA. Arrows point to pimonidazole negative, pS6 p4E-BP1 positive regions. Scale bar, 200 µm.
